# National Dental Association, Southern Branch

**Published:** 1898-07

**Authors:** C. L. Alexander

**Affiliations:** Charlotte, N. C.


### National Dental Association, Southern Branch.

Charlotte, N. C., May 28th, 1898.

To the Editor of THE DENTAL REGISTER.

DEAR SIR:—

Judging from the letters received, it is evident that there should be a wider diffusion of information in the South regarding the National Dental Association and its Branches. In many of the States the law does not require a diploma as a prerequisite for license. These State Societies which admit to membership all licensed practitioners, *regardless* of *diploma*, should therefore bear in mind that only their graduate members are entitled to election as delegates to the National Association and its branches. It should be made very clear that according to the constitution these bodies accept as *new* members only *delegates* elected by ballot at a regular meeting of the *State Societies*, and also that delegates must be *graduates* in dentistry or have acquired the degree of M. D. or have entered the profession prior to Sept. 1875. The American and the Southern Dental Associations, did also, it is true, require graduation as a prerequisite for membership, but as they did not restrict their eligible applicants for membership to *elected delegates* from *State Societies*, this features should therefore be emphasized. The requirements for membership in a branch of the National must necessarily be the same as in the National itself, as membership in the former confers membership in the latter. The above applies both to qualifications and to dues, which are $5.00 in either case, but it should be borne in mind that if the dues are paid directly to the National Treasurer this does not pay dues in the Branch, but the payment of $5.00 to the Treasurer of the Branch cancels all financial obligations to the National Treasury for the ensuing meeting, because the Branch forwards to the National Treasurer three-fifths of the dues received. Payment of dues to the Branch therefore insures for *a single fee double membership*, with all the rights, privileges and benefits of *both* bodies, including the joint volume of transaction.

By request of the President of the Southern Branch,

C. L. ALEXANDER,

*Cor. Sec. So. Branch Nat. Dental Ass'n.*

